# Evaluating the Psychiatric Benefits of Formulating Acetaminophen With N-Acetylcysteine

**DOI:** 10.3389/fpsyt.2020.564268

**Published:** 2020-10-23

**Authors:** Ryan Serdenes, James Graham

**Affiliations:** ^1^PGY2 Psychiatry Resident, Temple University Health System, Philadelphia, PA, United States; ^2^Department of Psychiatry, Lewis Katz School of Medicine at Temple University, Philadelphia, PA, United States; ^3^Medical Director-Crisis Response Center, Temple University Health System, Philadelphia, PA, United States

**Keywords:** N-acetylcysteine, acetaminophen, suicide, overdose, consultation-liaison psychiatry, harm reduction, suicide prevention

Acetaminophen induced hepatotoxicity is one of the leading causes of acute liver failure globally, is responsible for ~20% of total liver transplant cases, and costs the United States healthcare system $1.06 billion annually (1–3). Despite this, acetaminophen remains one of the most ubiquitous medications in the US—about one quarter of US adults ingest acetaminophen weekly (4). This is especially concerning from a psychiatric perspective considering 52% of acetaminophen overdoses are estimated to be intentional, with some studies reporting up to 86% of cases are associated with suicidality (3, 5). In the United States, an estimated 55,000 patients present annually to emergency departments with suicide attempts via acetaminophen ingestion (6). Aside from antidote therapy, only per purchase quantity restrictions have been associated with decreased incidence of completed suicides via acetaminophen overdose (7). At the time of writing, only two publications exist discussing the merits of dual formulation acetaminophen and N-Acetylcysteine (NAC) in reducing medical sequalae of overdose; however, neither apply a psychiatric viewpoint to the issue (8, 9).

NAC is on the *World Health Organizations List of Essential Medicines*, which is reserved only for the safest and most efficacious medications within healthcare (10). Besides acetaminophen overdose, NAC is indicated as a mucolytic and has been commercially available at affordable prices to the public for off-label use. NAC is tolerated well in high doses and has sparse inconsistent reports documenting severe adverse effects (11). In the context of acetaminophen induced hepatotoxicity, NAC therapy replenishes hepatic glutathione required for conjugation and neutralization of acetaminophen's toxic metabolite NAPQI. With timely NAC therapy, mortality rates are reduced to 0.4% and the NNT for death prevention in the context of fulminant hepatic failure is estimated to be 4 (12, 13). Several research studies have also established similar safety and efficacy profiles for both oral and IV NAC protocols (14).

Considering this, it seems reasonable that dual formulation acetaminophen/NAC should be an optional preparation of acetaminophen containing products, especially for patients at increased risk for suicide. This concept is not without precedent, as buprenorphine is prepared with its antidote naloxone to prevent medical misuse. Patients who have overdosed would be functionally NAC loaded prior to formal medical intervention in the emergency setting. Aside from the likely mortality benefit from dual formulation, hospital length of stay may be shortened, expediting patient placement on inpatient psychiatry and subsequent treatment if indicated. Additionally medical illness is a well-documented risk for psychiatric disease, so stymying medical consequences of overdose may lessen the severity of psychiatric symptoms (15). Finally, since overdose remains one of the most common methods in both impulsive and planned suicide attempts—it's possible that patients may judge the combination preparation as an inferior method of suicide and turn to other less toxic means of ingestion, especially if they lack a medical background (16).

However, conceptualizing a dual formulation prompts several practical considerations such as optimal NAC to acetaminophen dosing ratio, drug pricing, and side effects. While the theoretical efficacy of such a preparation seems obvious, this would need to be confirmed in clinical studies. Additionally, although NAC is offered at affordable commercial prices, the process of compounding NAC with acetaminophen may be non-intuitive and require additional manufacturing resources, resulting in an increased price. Moreover, even though NAC is exceptionally well-tolerated in terms of side effects, the consequences of chronic NAC ingestion would need to be investigated further to assure safety. The prospects of alternative antidote coformulation with methionine may be contemplated; however, high-dose methionine has been demonstrated to induce metabolic acidosis and chronic use may be cardiotoxic as homocysteine is a significant methionine metabolite (17).

Paternalism is commonplace in the history of medical practice and public health—evidence of it can even be found in fluoridated tap water, which is a World Health Organization recommendation for the prevention of dental caries (18). So considering the economic, psychiatric, and medical impacts of overdose related acetaminophen induced hepatotoxicity, it seems rational that this subject should get, at minimum, equal attention as dental caries. In summary, there is sufficient evidence and necessity to justify providing an optional Acetaminophen/NAC formulation—a idea public health officials and drug manufacturers should seriously consider.

## Author Contributions

All authors listed have made a substantial, direct and intellectual contribution to the work, and approved it for publication.

## Conflict of Interest

The authors declare that the research was conducted in the absence of any commercial or financial relationships that could be construed as a potential conflict of interest.
